# Automated Generation of Reliable Blood Velocity Parameter Maps from Contrast-Enhanced Ultrasound Data

**DOI:** 10.1155/2017/2098324

**Published:** 2017-05-30

**Authors:** Benjamin Theek, Tatjana Opacic, Diana Möckel, Georg Schmitz, Twan Lammers, Fabian Kiessling

**Affiliations:** ^1^Institute for Experimental Molecular Imaging, RWTH Aachen University Clinic and Helmholtz Institute for Biomedical Engineering, Pauwelsstr. 30, 52074 Aachen, Germany; ^2^Department of Medical Engineering, Ruhr-University Bochum, Universitätsstr. 150, 44801 Bochum, Germany

## Abstract

**Objectives:**

The purpose of this study was the automated generation and validation of parametric blood flow velocity maps, based on contrast-enhanced ultrasound (CEUS) scans.

**Materials and Methods:**

Ethical approval for animal experiments was obtained. CEUS destruction-replenishment sequences were recorded in phantoms and three different tumor xenograft mouse models. Systematic pixel binning and intensity averaging was performed to generate parameter maps of blood flow velocities with different pixel resolution. The 95% confidence interval of the mean velocity, calculated on the basis of the whole tumor segmentation, served as ground truth for the different parameter maps.

**Results:**

In flow phantoms the measured mean velocity values were only weakly influenced by the pixel resolution and correlated with real velocities (*r*^2^ ≥ 0.94, *p* < 0.01). In tumor xenografts, however, calculated mean velocities varied significantly (*p* < 0.0001), depending on the parameter maps' resolution. Pixel binning was required for all in vivo measurements to obtain reliable parameter maps and its degree depended on the tumor model.

**Conclusion:**

Systematic pixel binning allows the automated identification of optimal pixel resolutions for parametric maps, supporting textural analysis of CEUS data. This approach is independent from the ultrasound setup and can be implemented in the software of other (clinical) ultrasound devices.

## 1. Introduction

Over the last decades tumor heterogeneity has gained more and more attention in cancer research [1–3]. The highly complex and variable process of cancer evolution causes the development of a wide variety of genetically and phenotypically different cancer cell subclones in tumors [2, 4, 5]. Since tumor heterogeneity is recognized as an indicator for poor clinical prognosis, increasing efforts are spent on characterizing the spatial and temporal heterogeneity of tumor phenotypes [6–8]. In this regard, medical imaging techniques are increasingly used to noninvasively acquire tomographic images and to assess intratumoral heterogeneity [9–11], to improve disease diagnosis, prediction of therapy response, treatment monitoring, and prognosis [7, 12, 13].

Contrast-enhanced (CE) imaging strongly supports anatomical imaging when it comes to the detection of therapy responses [14, 15]. However, most previous studies on texture analysis and radiomics are based on morphological features and not on functional (vascular) data [12, 13, 16, 17].

Ultrasound (US) is an inexpensive and widely available imaging modality which is highly suitable to determine anatomical, functional, and molecular parameters of tumors [18]. Postprocessing of contrast-enhanced ultrasound (CEUS) data, that is, the calculation of functional parameters based on time-intensity curves (TIC), is used to generate functional parameter maps to assess intratumoral heterogeneity [19, 20]. The quality of these parameter maps depends on the spatial resolution, the ultrasound frequency, the contrast agent, the imaging system, and the technique used for image acquisition. A high spatial resolution, for example, pixel-wise analysis, which is desired by the user, is usually accompanied by low signal-to-noise ratios (SNR), as the likelihood of a sufficient amount of microbubbles (MB) is reduced in smaller resolution cells. In addition, smaller segmentations are more susceptible to motion artifacts, which further reduces the SNR and complicates reliable curve fitting to the data. Therefore, it is important to individually validate parameter maps.

For that reason, we developed a postprocessing approach that allows the automated generation of validated functional parameter maps from destruction-replenishment CEUS data by systematic pixel binning. We propose that future studies should implement such validation steps in their analysis to ensure the reliability and robustness of their parameter maps.

## 2. Materials and Methods

### 2.1. Flow Phantom Experiments

A gelatin phantom containing a single channel of 0.96 mm in diameter was prepared for MB velocity measurements. In-house produced polymeric (poly-butyl cyanoacrylate; PBCA) MB with a mean diameter of 2 *μ*m were used as US contrast agent [21]. Using a syringe pump and a MB concentration of 5*∗*10^8^ MB/ml, several destruction-replenishment cine loops (*n* = 4) were recorded at various flow velocities ranging from 0.038 mm/s to 1.21 mm/s. B-mode images were calculated from the raw radio-frequency (RF) CEUS data and analyzed as described below.

### 2.2. Mouse Tumor Model

All animal experiments were approved by the governmental ethics approval committee. To induce tumor growth 4*∗*10^6^ cells of either A431, MLS, or A549 origin were injected into the right flank of CD-1 nude mice (Charles River, Sulzfeld, Germany) (*n* = 3 for each group). When the tumors reached a size of 5–8 mm in diameter, the mice were subjected to CEUS imaging. After CEUS imaging the mice were sacrificed and tumors were removed for histological analysis (see Supplemental Material, available online at https://doi.org/10.1155/2017/2098324).

### 2.3. Contrast-Enhanced Ultrasound Imaging

During the whole imaging procedure, the mice were kept under continuous anesthesia using 2% v/v isoflurane. Each mouse received a bolus injection of 50 *μ*l contrast agent (2*∗*10^8^ PBCA MB/ml) via a tail vein catheter. The injection phase and the subsequent destruction-replenishment sequence were recorded at 50 frames per second, using the MS550D transducer, operating at 40 MHz, connected to the Vevo 2100 System (FUJIFILM VisualSonics Inc., Toronto, Canada). The B-mode images were directly computed from RF-data and analyzed as described below.

### 2.4. Calculation of Blood Flow Velocities

A region of interest (ROI), that is, tumor or channel, was manually selected for further analysis. The calculation of the mean blood flow velocity was based on a slightly modified version of the destruction-replenishment model by Wei et al. [22]. The signal intensity, generated by the contrast agent reentering the imaging slice after a destructive pulse, was modeled by the following exponential function:

(1) $$\begin{array}{c} y\left(\;t\;\right)=A\left(\;1-e^{-\beta t}\;\right)+c, \end{array}$$

where *A* is the amplitude, *β* is the rate constant determining the steepness of the rise, and *c* is an additional factor to shift the function. The time constant *τ* is the inverse of the rate constant *β* and has to be multiplied with the thickness of the imaging plane* d* to obtain the velocity *v*.

(2) τ=1β,v=τd.

The curve fitting was performed using MATLAB R2015a (MathWorks, Natick, USA). The 95% confidence interval of the mean blood flow velocity was used to evaluate the reliability of the parametric perfusion maps.

### 2.5. Generation and Validation of Parametric Maps

Systematic pixel binning (*n*^2^ × *n*^2^; *n* = 1–8), by averaging the signal intensities, resulted in several datasets with differing spatial resolution. Similar to the algorithm described above, an exponential function was fitted to the TIC of each individual pixel of the ROI in all datasets. To exclude pixels with low SNR, exclusion criteria were introduced (see Supplement Figure  1). In accordance with the imaging data, our algorithm calculated parametric velocity maps with a resolution ranging from 1 × 1 pixel to 256 × 256 pixels (*n*^2^ × *n*^2^; *n* = 1–8). With the imaging setup used, the reconstructed images had a pixel size of 22 *μ*m × 22 *μ*m and 55 *μ*m × 22 *μ*m for the phantom measurements and the in vivo measurements, respectively. For validation purposes, the postprocessing algorithm calculated for each parametric velocity map a mean flow velocity, by averaging the individual velocities of the ROI. The reliability of the parameter maps was validated by comparing the average velocity of each single parameter map with either the preadjusted velocity of the syringe pump (phantom experiments) or the velocity calculated from the whole tumor segmentation (in vivo experiments). Only when the average velocity of the parameter map lies in the 95% confidence interval of the velocity calculated from the whole tumor segmentation, the parameter map was regarded as reliable.

### 2.6. Statistical Analysis

The nonparametric Spearman correlation coefficient and coefficient of determination of preadjusted and calculated MB velocities as well as the one-way ANOVA analysis of the in vivo data were determined using GraphPad Prism 5 (GraphPad Software, La Jolla, USA).

## 3. Results

### 3.1. Flow Phantom Experiments

**Figure 1:**
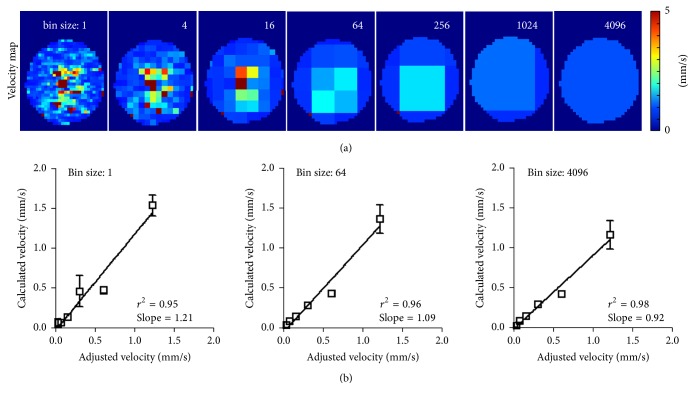
Flow phantom measurements. Parametric maps of different resolutions were calculated and color-coded as shown in (a). At higher resolution, a laminar flow profile can be observed. These measurements were performed at different flow speeds and used to calculate the mean flow velocity. The correlation (*p* < 0.01) of calculated mean flow velocity and preadjusted flow velocity are exemplarily shown for a pixel-wise analysis, a binning size of 64, where 8 × 8 pixels were binned, and a binning size of 4096, where the whole ROI is covered by a single segmentation in (b). It proves the independence of the pixel size and mean flow velocity of the whole ROI at ideal conditions, as well as the functionality of our algorithm to calculate flow velocities. The correlations for the other binning sizes are shown in Supplement Figure  2.

**Figure 2:**
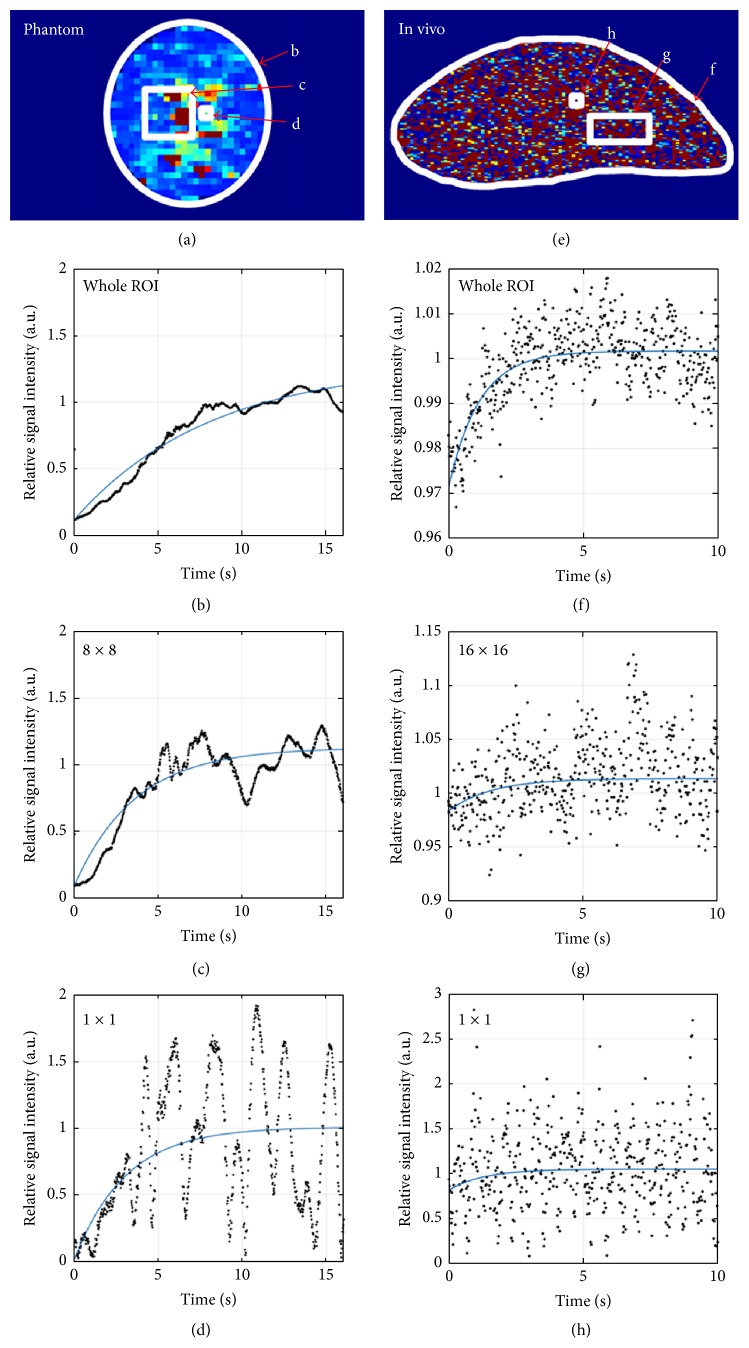
MB velocity map and TIC of a phantom and A431 tumor measurement. The exemplary velocity maps of a pixel-wise analysis are depicted in (a) and (e). The representative TIC of the segmentations demarked in white are presented in (b)–(d) for the phantom measurement and in (f)–(h) for the in vivo measurement.

Irrespective of the pixel resolution, the mean MB velocity of all parameter maps in Figure 1(a) was similar. As shown in Figure 1(b) and Supplement Figure  2, the calculated mean flow velocities correlated very well with the preset velocities of the syringe pump, resulting in *r*^2^-values of at least 0.94 and slopes of the linear regression curve close to the ideal value of 1. Figures 2(a)–2(d) show exemplary TIC of differently sized segmentations and the resulting exponential curve fit. An increase in signal fluctuations with decreasing segmentation size was found, which, however, still resulted in a reasonably good exponential curve fit for the pixel-wise analysis (Figure 2(d)).

### 3.2. In Vivo Experiments

The applicability of our algorithm was tested in three tumor xenograft models, which are known to express different patterns of angiogenesis [23]. While A431 tumors are highly angiogenic and show many small immature vessels homogeneously distributed over the entire tumor, MLS tumors are heterogeneous with immature and mature regions. A549 tumors are the most mature and least vascularized ones with many large vessels in the periphery [23]. For all tumor models the best curve fit was obtained when the whole tumor area was segmented. In this context, A549 (0.09 ± 0.02 mm/s) and A431 (0.1 ± 0.05 mm/s) tumors showed higher MB velocity values than MLS tumors (0.07 ± 0.01 mm/s; Supplement Figure  3).

**Figure 3:**
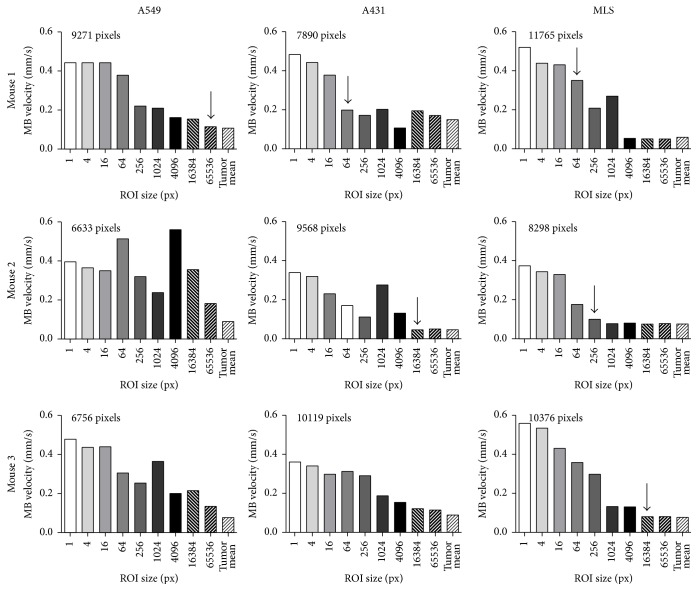
Parametric perfusion analysis of mouse tumors. Graphs of nine individual in vivo measurements displaying the calculated mean MB velocity of different pixel resolution. The arrow indicates the parameter map with the best pixel resolution, having a mean MB velocity falling into the 95% confidence interval of the MB velocity calculated for a single segmentation, which is covering the whole tumor (tumor mean). The high variability in between the measurements shows the need for an individual assessment of each tumor. The number of pixels on the top of each graph displays the size of the tumor segmentation.

When the parameter maps were analyzed, increasing mean velocities were observed with increasing resolution of the parameter maps (*p* < 0.0001). Apparently, the background noise in TIC of smaller segmentations was misinterpreted by the software as replenishment effect (Figures 2(e)–2(h)). Furthermore, the number of pixels, which needed to be binned to obtain reliable parameter maps, varied between individual measurements and tumor models (Figure 3). The number of pixels which had to be binned to generate reliable parameter maps was highest in A549 tumors, followed by A431 and MLS tumors. For some of the measurements none of the parameter maps was identified as reliable.

**Figure 4:**
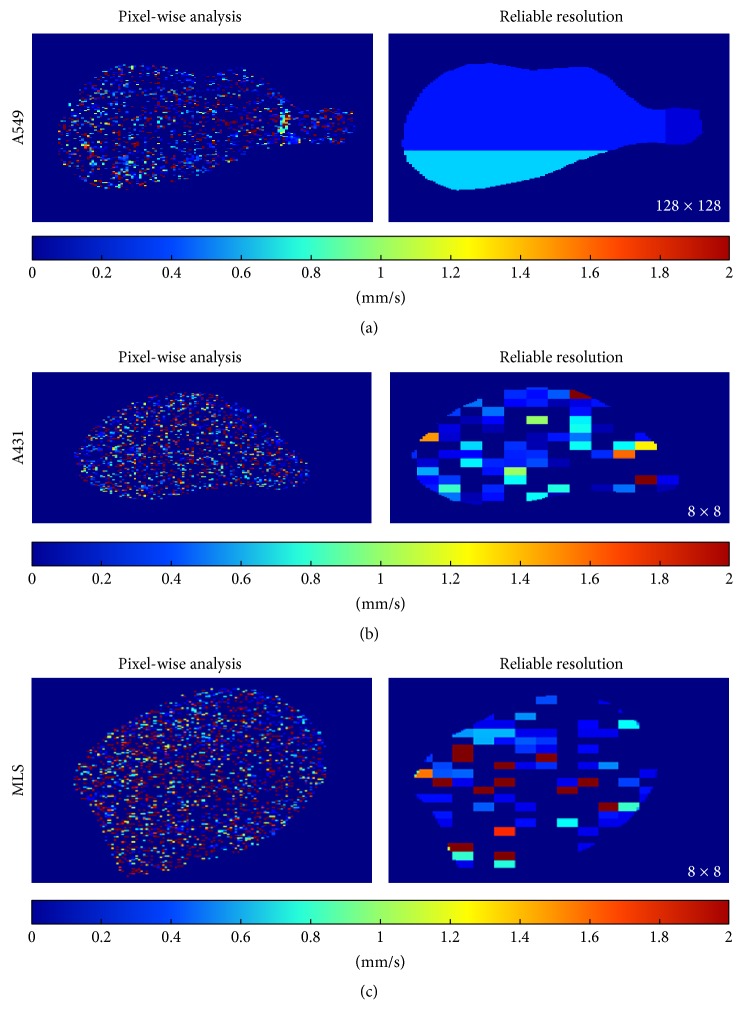
Exemplary parametric MB velocity maps for each tumor model. The left panel presents a parametric map which is based on a pixel-wise analysis. The right panel presents the validated parametric map of our analysis algorithm. For the A549 tumor 128 × 128 pixels had to be binned. The parameter map enables the identification of a region of higher MB flow velocities in the lower periphery (a). The reliable parameter map of the A431 tumor, with a binning of 8 × 8 pixels, shows a homogeneously distributed vascular network with relatively low MB velocities (b). Also in this MLS tumor a binning of 8 × 8 resulted in a reliable parameter map. In this context, regional differences and the more heterogeneous distribution of velocities as well as the larger spectrum of different velocity values can clearly be identified.

**Figure 5:**
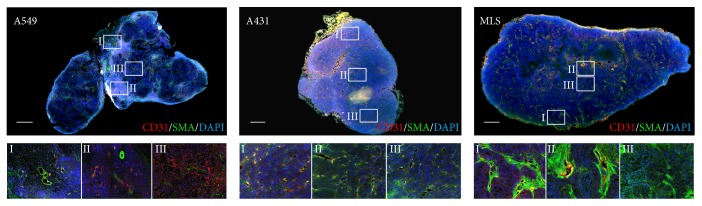
Representative immunohistochemistry images of A549, A431, and MLS tumors. The A549 tumor shows larger, more mature vessels in the tumor periphery, as it can be seen on the magnified image sections I and II. In contrast, the core shows a lower degree of vascularization with smaller vessels. A431 tumors have small immature vessels homogeneously distributed throughout the tumor, as it can be seen on the magnified image sections from the tumor periphery and core. MLS tumors present a more heterogeneous vessel network. This tumor model presents bigger vessels throughout the tumor (I and II). However, directly next to these highly vascularized areas, regions of lower vascularization can be observed (II and III). The scale bars correspond to 600 *μ*m.

Without binning, hardly any regional differences in the flow velocities were observed in the parametric maps and, thus, all three tumor models looked similar (Figures 4(a), 4(b), and 4(c); pixel-wise analysis). However, differences in between the tumor models become apparent if pixel binning is performed (Figures 4(a), 4(b), and 4(c); reliable resolution). For A431 and MLS tumors the parameter maps of higher resolution (8 × 8 pixels) were in good agreement with data from literature and our own findings, about the vascular composition of these tumors: the MB velocity values in A431 tumors showed a lower variability than in MLS tumors and a relatively homogeneous distribution (Figure 4(b)). As indicated in Figure 4(c) the vascular network of the MLS tumor was more heterogeneously distributed with many unperfused pixels and pixels with high velocity values. These results are in line with the histological images, A431 tumors have a relatively dense network of small immature vessels throughout the tumor, and MLS tumors show a more heterogeneous network of small and large vessels (Figures 5(b) and 5(c)). Due to the high binning rate in A549 tumors (128 × 128 pixels), structural characteristics of the vasculature could hardly be assessed (Figure 4(a)). A larger vessel, which can be seen in the pixel-wise analysis on the right side of the tumor, is masked by the high binning rate. However, in line with the presence of larger vessels in the tumor periphery that were found in histology (Figure 5(a)), the reliable parameter map presented in Figure 4(a) shows a higher MB velocity in the lower periphery of the tumor.

## 4. Discussion

Our results show that CEUS parameter maps of in vivo measurements are strongly influenced by noise, pointing to the need for their validation. We show that if this is done on an individual basis the maximal pixel resolution of a reliable parameter map can be obtained. In our study, it would have been desirable to additionally compare our results to a gold standard of blood flow velocity; however, imaging tumor blood flow velocity with other imaging techniques, such as DCE MRI or DCE CT, is difficult as well and often not possible at a quantitative level. In addition, this is complicated by image registration problems which occur when trying to image the same regions of interest. Furthermore, in contrast to the phantom measurements, the in vivo measurements cannot be compared to and validated by a priori known values. However, if the calculations are reliable, all parameter maps of different pixel resolutions have approximately the same average velocity, as in case of the phantom experiments. The most reliable mean tumor blood flow velocity calculation for each measurement is based on the TIC of the whole tumor segmentation. This measurement is the best available control to validate the different in vivo parameter maps. Defining the 95% confidence interval of each individual ground truth as validation criterion links the goodness of the ground truth measurement directly to the parameter map. Thus, our approach takes the variability of individual measurement into account and does not use a fixed number for pixel binning as it is performed in other studies [19, 20].

While A431 tumors are highly angiogenic and show many small immature vessels homogeneously distributed over the entire tumor, MLS tumors are heterogeneous with immature and mature regions. A549 tumors are the most mature and least vascularized ones with larger vessels in the periphery [23]. With respect to the intratumoral vessel distribution, the A549 tumor model is the most heterogeneous one. At the same time, the A549 tumor model presents the highest binning rates. We expect that largely unperfused areas in the tumor core, which are apparently not excluded by our exclusion criteria as well as the most restrictive 95% confidence interval, prevent parameter maps of higher resolution to be defined reliably.

To improve the applicability of our algorithm the following limitations should be addressed in future studies. First, our binning approach is based on a chessboard-like segmentation, which does not consider the shape and position of the tumor. Depending on the location and size of the individual segmentations they might suffer from a partial volume effect, compromising the quality of the respective parameter map. Mathematical optimization of the location, shape, and size of the segmentations could help to overcome this issue. This would potentially result in a finer segmentation and resolution in highly perfused areas and lower resolution in avascular areas. Second, the exclusion criteria, to identify pixels with low SNR, can be refined. Third, alternative ground truth definitions, for example, using a fixed percentile, can be tested. Fourth, preprocessing the data, to account for the complex noise statistics, might reduce the reliable resolution of the parameter map. Fifth, a continuous infusion of the contrast agent, instead of a bolus injection, would enable several destruction-replenishment sequences, reducing the variability caused by the measurement. Sixth, more sophisticated fitting algorithms should be tested to reduce systematic errors which might have been introduced by the model used in this study. Seventh, all experiments were performed with a preclinical US device using experimental MB. However, neither the use of a clinical US device nor of clinically approved MB should influence our software-based analysis strategy. As in clinical US images pixel sizes are larger and MB specific scan modes are available, we even expect better performance due to higher SNR.

Overall, our results show that highly spatially resolved parameter maps, obtained from high-frequency CEUS, need to be validated. Systematic pixel binning can help to identify the pixel resolution to generate reliable parametric maps for each individual measurement. We want to stress the fact that with pixel binning the reliability of the parameter map is improved, but spatial information is lost. Alternative binning methods, with variable resolution in different areas of the ROI, as well as more extensive preprocessing of the US data, to remove noise, should be tested in future studies to improve the resolution of reliable parameter maps. As this method is independent of the used equipment, it can be implemented into other (clinical) US software packages to validate functional parameter maps. Reliable parameter maps will better support textural analysis of CEUS data and thus may be capable of improving diagnosis, prediction of therapy response, treatment monitoring, and prognosis.

## Supplementary Material

Supplement Material 1: Immunohistochemistry.
Supplement Figure 1: TIC and exclusion criteria.
Supplement Figure 2: Flow phantom measurements.
Supplement Figure 3: Mean tumor blood flow velocities.
