# Supplementary material for: Automated Generation of Reliable Blood Velocity Parameter Maps from Contrast-Enhanced Ultrasound Data
Source: Contrast Media Mol Imaging. 2017 May 30;2017:2098324. doi: 10.1155/2017/2098324 (PMC5612675; doi:10.1155/2017/2098324)
Supplement: Supplementary file 1 — Supplement Material 1: Immunohistochemistry. Supplement Figure 1: TIC and exclusion criteria. Supplement Figure 2: Flow phantom measurements. Supplement Figure 3: Mean tumor blood flow velocities. [file 2098324.f1.docx]

**Supplemental Material**

***Immunohistochemistry***

Tissue sections of 8 µm thickness were cut using a Leica CM3050S (Leica Biosystems, Wetzlar, Germany) cryotome. The cryosections were stained using CD31 (5 µg/ml; BD Biosciences, Heidelberg, Germany) and αSMA-biotin (6 µg/ml; Progen, Heidelberg, Germany) antibodies for 1h at room temperature. Three washing steps of 10min each, using PBS, followed. Then, the cryosections were incubated with the secondary antibodies Cy3-conjugated Donkey anti-rat against CD31 (2.8 µg/ml) and Cy2-conjugated Streptavidin against αSMA-biotin (8.5 µg/ml) for 45 min (both from Dianova, Hamburg, Germany). The cell nuclei were stained using DAPI (5 µg/ml; Merck, Darmstadt, Germany) applying an incubation time of 10 min at room temperature. After 3 additional washing steps the cryosections were covered with Mowiol and coverslips. Automated whole-slide fluorescence microscopy was performed using the Vectra 3.0 Imaging System (PerkinElmer, [Waltham](https://de.wikipedia.org/wiki/Waltham_%28Massachusetts%29), [Massachusetts](https://de.wikipedia.org/wiki/Massachusetts), [USA](https://de.wikipedia.org/wiki/Vereinigte_Staaten)). The histological images were assessed by a radiologist (FK) and biologist (BT). The descriptive findings of both examiners were in good agreement, and in line with the findings published by Ehling et al. [23]. The criteria for heterogeneity were of qualitative nature, describing the vessel size, maturity and their distribution in the tumor.


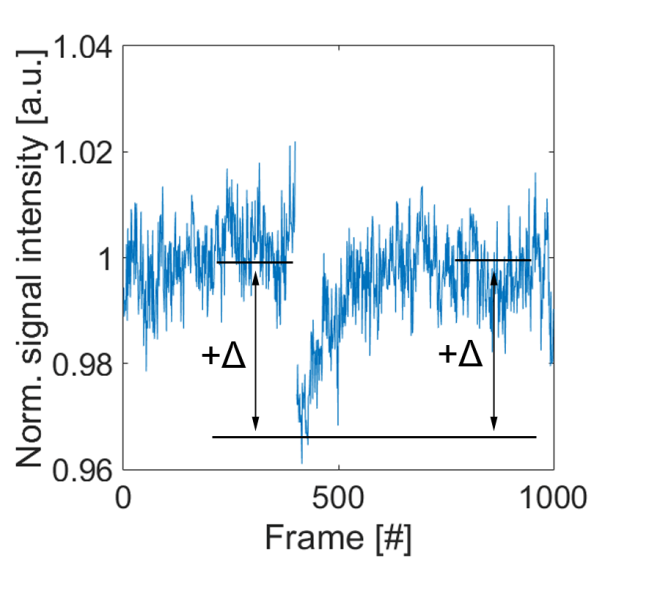


***Supplement Figure 1:*** *Time intensity curve of a destruction replenishment sequence, displaying the mean signal intensity of the whole tumor over time. Based on the shape of the destruction replenishment curve two exclusion criteria were defined to reduce the number of low SNR pixels in the analysis. The signal difference before and after MB destruction, as well as at the end of the sequence and after MB destruction, must be positive. Only if both conditions are fulfilled the respective pixel or segmentation will be analyzed.*


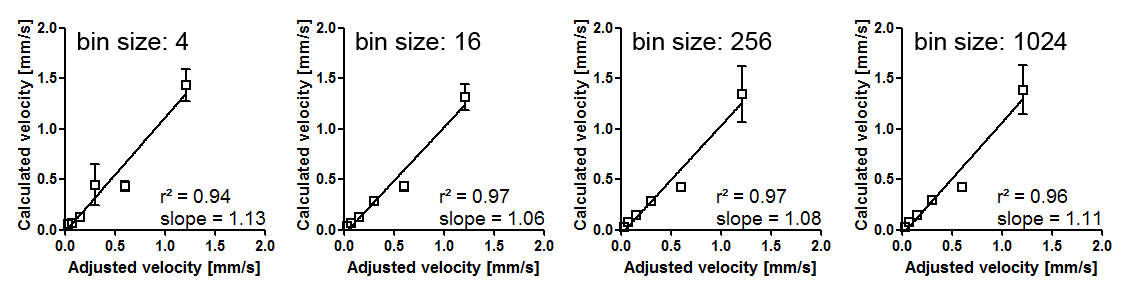


***Supplement Figure 2:*** *Flow phantom measurements. Correlations (p<0.01) of calculated mean flow velocities and pre-adjusted flow velocities are shown for binning numbers of 4 (2x2 voxels), 16 (4x4 voxels), 256 (16x16 voxels), and for 1024 (32x32 voxels). In line with data presented in Figure 2, good correlations (r² ≥ 0.94) are observed.*

***
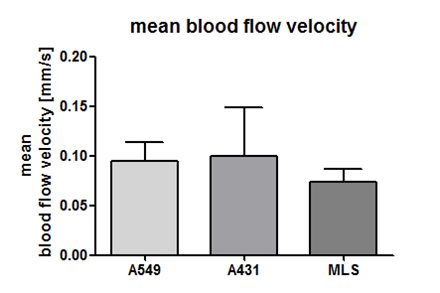
***

**Supplement Figure 3:** *Mean MB velocities calculated on the basis of whole tumor segmentations. Each column presents the mean ± SD of the three tumors of each model.*
